# Transient Heat Transfer Characteristics in a Flat Plate Heat Sink with Mini-Channels for Cooling High Heat Flux IGBT

**DOI:** 10.3390/mi13091417

**Published:** 2022-08-28

**Authors:** Changnian Chen, Haoran Zhao, Chaoyu Liu, Jian Chen, Chunyang Liu, Tiezhu Zhang, Weiping Gong

**Affiliations:** 1 School of Energy and Power Engineering, Shandong University, Jinan 250061, China; 2 Optics & Thermal Radiation Research Center, Shandong University, Qingdao 266237, China; 3 School of Electrical Engineering, Shandong University, Jinan 250061, China; 4 Shandong Guochen Equipment Installation Co., Ltd., Jinan 250305, China; 5 Taishan Fiberglass Inc., Taian 271000, China

**Keywords:** transient heat transfer, mini-channel, flat plate heat sink, high heat flux, IGBT

## Abstract

Effective cooling of a high heat flux IGBT electronic system is highly related to system efficiency and safety. A flat plate heat sink was designed to experimentally investigate the transient heat transfer characteristics of IGBT cooling. It is made of aluminum with 20 mini-channels of 249 mm × 3 mm × 4 mm dimensions, which were manufactured by milling machines and melt inert gas (MIG) welding technology to ensure no deformation. Experiments were conducted using deionized water at atmospheric pressure with flow rates of 3.2–9.5 L/min and heat fluxes of 104–347 W/cm^2^. It was found that instantaneous start-stop and transient heating power variation might cause IGBT failure, especially under low Reynolds and Nusselt number conditions. The temperature rise rate and cooling rate vary with different system parameters. Heating rate can be reduced by high flow rate due to local subcooled boiling. The concept of respond time (RT) based on the piecewise function is suggested to evaluate the influence of transient condition on heating rate. Analysis of flow fluctuation indicated that it would not be a threat to the system except for in extreme cases. These findings provide a reference for the considerations of the design and manufacture of IGBT cooling flat plate heat sinks with mini-channels.

## 1. Introduction

With the development of the insulated gate bipolar transistor (IGBT) towards high power and high integration, which is widely used in ultrahigh voltage direct current transmission (UHVDC) electricity and kinds of control fields, there are great improvements in structure and performance [1,2,3]. However, the problem of heat generation is becoming increasingly prominent synchronously, and the requirements for heat dissipation are a matter of great concern for researchers [2,3,4,5]. As the IGBT chip is the core functional device that generates heat, the accumulation of heat will seriously affect the working performance of the electronic device due to the high switching frequency and large through current. Thus, if the heat generated is not taken in time the temperature of integrated IGBT packaging surface will increase substantially, which is very dangerous for devices and systems. When the temperature exceeds the tolerance range of electronic components (usually no more than 80 centigrade) that leads to the IGBT failure or damage—even system paralysis [2,6,7,8]. Therefore, it can be seen that ensuring fast and effective heat dissipation of the IGBT module is very important for its safe and reliable operation.

Researchers so far developed diverse techniques to remove heat generated by high power electronic devices and proposed numerous improvement measures to enhance heat transfer in pipes or flow channels [9]. Traditional applications of heat dissipation, including natural air cooling, forced air cooling, natural water cooling, forced water cooling, phase change heat transfer, etc. are widely used for cooling electronic devices and systems. Kang [10] studied the air cooling effect for a single IGBT module. The dimensions of heat sink and the IGBT module are 236 mm × 230 mm and 162 mm × 122 mm, respectively. It is proven that heat from power of up to 2 kW can be removed effectively when the air velocity is 0.25 m^3^/s. Zhang et al. [11] analyzed different forced air cooling schemes, and they concluded that the temperature rise rate and cooling rate were basically the same when the heat sink was considered as a homogeneous body. Vasiliev et al. presented a new loop heat pipe (LHP) technology to cool systems for high-power IGBT elements, and they achieved a nominal capacity of 900 W for a steady-state condition and more than 900 W for a periodic mode of operation at a temperature level below 100 °C [12]. Lu et al. proposed a rectangular heat pipe radiator with a parallel heat flow structure, and they used it to cool down two 1700 V/1000 A IGBTs by a mean temperature of 65 °C, which of course needs a very large air flow rate to act effectively [13]. Actually, heat pipe technology can really achieve a good cooling effect, but its hot end is still limited by air cooling conditions [11,14]. Although direct air cooling is a mature technology and has simple equipment for operation, even it can achieve good cooling effect sometimes, but its practical application tends to be limited by heat capacity against the increasing requirements of high heat flux dissipation. Subsequently, researchers turned to research using liquids as coolant, including water, glycol, HFCs, HFEs, liquid metal and nanofluid with multifarious additives etc., which lead to a higher heat transfer coefficient and capacity [14,15,16,17]. Lee [18] presented a novel approach to optimize the pin array design of IGBT considering liquid-cooled conditions, which achieved a high heat power dissipation of 1200 W and kept the maximum junction temperature under 100 °C. It proved a competitive advantage of liquid cooling through the results that the maximum temperature variation did not exceed a 1 °C increase. Valenzuela et al. [19] developed a liquid-cooled technique to remove the heat from semiconductor modules, which allowed dissipation of up to 300 W/cm^2^ with an approach temperature difference of less than 50 °C. It showed that the given technology reduces heat flow barriers while maintaining the package mechanical properties. Schulz-Harder et al. [20] concluded that the direct liquid cooling of base plates gave the best results; and stacked liquid-cooled modules introduced the possibility of switching high power in a minimum volume. Wang et al. [21] found that the thermal resistance of junction to a heat sink could reduce more than 50% by direct liquid cooling due to the elimination of the thermal grease layer. Therefore, the active and passive temperature fluctuations are significantly reduced, which can improve the reliability and life of the module. Lim and Lee [22] proposed a two-phase counter flow mini-channel heat sink with interlocking double layer structure and confirmed that compared with the traditional one-way heat sink, the counter flow heat sink can obtain a more uniform temperature distribution, so that counter flow configuration can improve the uneven temperature distribution. 

In terms of cooling structure, mini/micro-flow channels attracted much attention on account of their compactness in structure and high efficiency in heat transfer [23,24,25,26] compared with macro-channels and various special-shaped cooling channels [27,28,29]. As for coolant and its phase state for cooling purpose, researchers used various working fluids, with either single, phase change, or other kinds of additives for heat transfer enhancement, according to specific application [30,31,32,33,34,35,36,37,38,39,40]. Generally, convective heat transfer cannot meet the heat dissipation of high flux IGBT due to its low thermal conductivity [31]. Campbell et al. [32] and Dalkilic et al. [33] used the low latent heat refrigerant R134a of flow boiling heat transfer to cool electronic devices. The results show that the cooling effect of this cooling mode was significantly better than that of the ordinary convection heat transfer without phase change. Ghodsinezhad et al. [34], Arani et al. [35], Bahiraei et al. [36], Khetib et al. [37], Ghachem et al. [38] and Omri et al. [39] introduced several nanofluids with different additive particles, including water-based Al_2_O_3_, CuO, SiO_2_, and CNT, etc., to further improve the thermal conductivity of working fluid. They achieved better heat transfer enhancement to varying degrees. Additionally, many researchers [40,41] used liquid metals as coolant to enhance heat transfer successfully, benefiting from the great thermal conductivity of metals. Recently, Wu et al. [42] investigated the heat transfer characteristics of liquid metals in micro-channel heat sinks for high-density heat dissipation. They took the alkalis as objects of study, covering sodium (Na), potassium (K), sodium-potassium alloy (Na-K), and lithium (Li), and reported a result of an approximate 75% enhancement in heat transfer coefficient. However, there are still some issues with nanofluids and liquid metals, such as the performance limitations due to base fluid, easy fouling or clogging channels, and potential low critical heat flux (CHF) because of a relative low boiling point.

Previous literature indicates that liquid cooling technologies with mini/micro-channels are more efficient for high-flux IGBT cooling. There are many special aspects that need to be considered for cooling of IGBTs used in high voltage transmissions, converter valves, and other high voltage level devices, such as an ultrahigh voltage direct current transmission (UHVDC), modular multi-level converter (MMC), and new energy converter (NEC), etc. Firstly, electrical insulation is the primary consideration in the heat dissipation system. Secondly, compactness of the heat exchanger is another necessary consideration due to the limited mount space. Thirdly, transient heat transfer analysis for high-flux IGBTs needs more attention in practice. In fact, existing studies mainly focus on the steady-state heat dissipation analysis for electronic devices according to the basic theory of heat balance and enhanced heat transfer technology. Transient heat transfer analysis is scarce and needs more work, which is directly related to device fatigue and safety [4,8,17]. Lastly, the cost-effectiveness of heat dissipation systems are always pursued because of the huge amount of IGBT usage.

Based on the analysis above, a compact flat plate heat sink with mini-channels for cooling a high heat flux IGBT was designed in this paper. Deionized water was selected as coolant, considering the electrical insulation, economy, and safety for actual operation. Experiments were carried out to investigate the transient heat transfer characteristics of IGBT cooling, especially for some off-normal situations, such as instantaneous start-stop, heating power variation, flow fluctuation, etc., which will seriously affect IGBT cooling and even pose a threat to the whole system. Moreover, the concept of respond time (RT) was proposed to characterize the influence of the situations mentioned above on system safety.

## 2. Heat Sink Design and Experimental System

### 2.1. Design of the Flat Plate Heat Sink with Mini-Channels

Considering the compactness in space and advantages of the enhanced heat transfer of mini/micro-channels for cooling electronic devices, a water-cooled flat plate heat sink with parallel mini-channels was designed, manufactured, and assembled as a test section to study the transient heat transfer characteristics of IGBTs under various off-normal conditions, also taking into account the following factors: Firstly, a mini-channel has a larger flow passageway and lower pressure drop than a micro-size structure, and it has an absolute advantage in heat transfer over macro-size structure [24,25,26]. Then, mini-size structure is relatively easier to machine and has a lower cost. Additionally, electrical insulation and low thermal resistance material must be considered. Aluminum (type 6063T6) was used to ensure better thermal conductivity, which is not as expensive as copper. Deionized water was used to ensure excellent insulation and avoid the danger of local dry-out compared with low boiling temperature coolant. 

The flat plate heat sink was manufactured by milling machines and melt inert gas (MIG) welding technology to ensure no deformation. The manufacturing process follows the following procedures: (a) selecting an aluminum blank with the appropriate size as two sides (up and down) of the heat sink; (b) milling the down blank plate to form the required channel structure; (c) welding the up plate into the down one to form sealed flow channels using MIG technology; and (d) keeping both sides heated simultaneously to eliminate stress and minimize aluminum plate deformation. 

**Figure 1:**
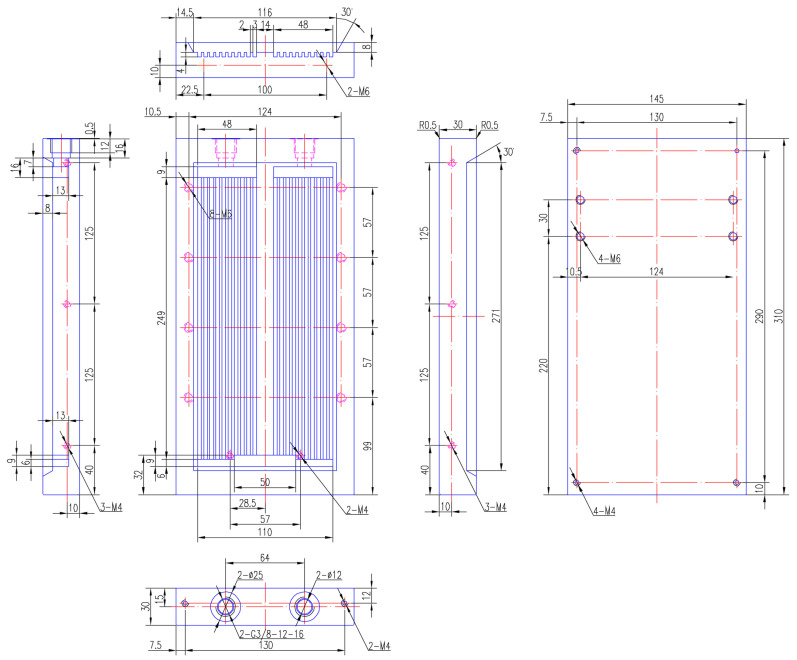
Structure and detailed geometric dimensions of the flat plate heat sink for IGBT cooling.

The overall geometric size of the flat plate heat sink designed is *L* × *W* × *H =* 310 mm × 145 mm × 30 mm. There are 20 flow channels in total, with a single flow channel size of *L_c_* × *W_c_* × *H_c_ =* 249 mm × 3 mm × 4 mm. The diameter of the inlet/outlet is 12 mm, which is determined by both structure and hydraulic factors. It is better to dig a larger hole to reduce the pressure drop, but this is limited to the side thickness of the aluminum plate, which is 30 mm, and space is left for sealing grooves and screw threads. See the specific parameters and detailed structure geometric dimensions in Table 1 and Figure 1. 

The hydraulic diameter of a single flow channel calculated by Equation (1) is about 3.43 mm, which belongs to a mini-channel according to the classification of channels by Mehendal et al. [43] and Kandlikar et al. [44], as shown in Table 2.

(1) $$D_{h}=\frac{4\cdot A_{c}}{L_{p}}=\frac{2\cdot a\cdot b}{a+b}$$

where *A_c_* is the cross section area of a single flow channel and *L_p_* is the wet circumference for a single channel. The parameters of *a* and *b* are the high and depth of a single channel, respectively. 

### 2.2. Experimental System Set-Up

**Figure 2:**
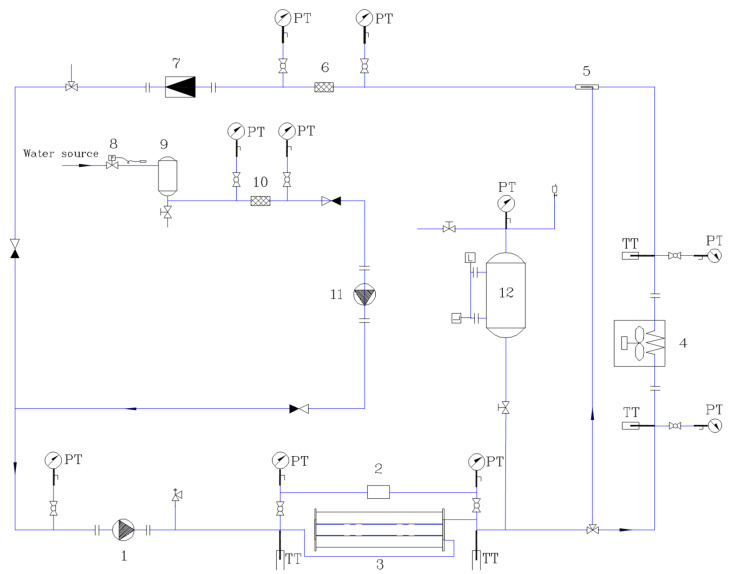
Schematic diagram of the experimental system with demineralized water treatment. 1: circulating pump, 2: differential pressure gauge, 3: testing platform, 4: air cooler, 5: water strainer, 6: main filter, 7: flow meter, 8: pressure regulator, 9: demineralized water tank 10-bypass filter, 11: supplementary water pump, 12: buffer tank, PT: pressure transducer, and TT: temperature transducer.

Experiments are performed in a main water pump closed cycle, as shown in Figure 2, accompanied by the demineralized water treatment open loop. The system mainly consists of water circulating the pump and meter, cooling circulation facilities, electric heating equipment, pressure stabilizing device, a testing platform, and a data acquisition instrument, with some auxiliary equipment for water purification, such as a strainer or filter and demineralized water tank, etc.

**Figure 3:**
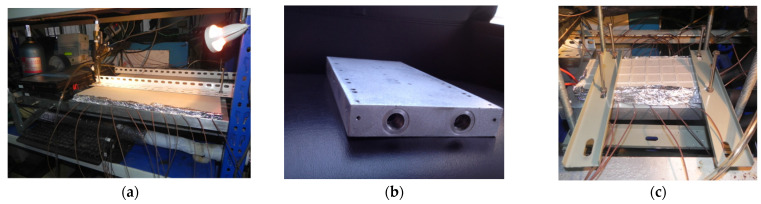
Photos of (**a**) experimental platform, (**b**) product of heat sink, and (**c**) test section rig.

Figure 3 shows several photos of the experimental system and heat sink rig. In the main cycle, water flows through the testing platform where the test section (heat sink) is installed, traps heat that needs to be removed, then is cooled down by the air cooler and flows back into the main circulating pump entrance, and the cycle continues. The demineralized water tank and its accessories can achieve the continuous deionization and electric regeneration of ion exchange resins. A buffer tank is used to provide stable pressure for the system. Various parameters, such as temperature, pressure, flow rate, current, and voltage are all collected by the Solartron isolated measurement pods (IMP) 3595 series (type 1A and 1C) data acquisition system, the technical parameters of which are listed in Table 3.

## 3. Experimental Details and Data Reduction

### 3.1. Experimental Details

**Figure 4:**
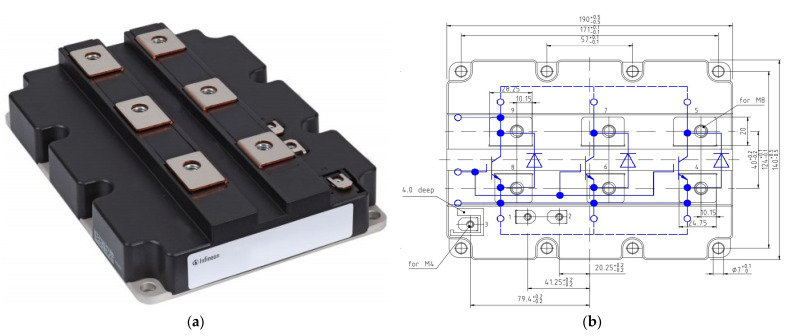
(**a**) Outline and (**b**) inward of IGBT module FZ1500R33HE3 targeted by the experiments.

The design of the flat plate heat sink in this paper aims for cooling a module IGBT (model FZ1500R33HE3) manufactured by Infineon^®^ (Neubiberg, Germany). The IGBT outline shape, inward structure, and geometric size, in detail, are shown in Figure 4, and the technical features are listed in Table 4 [45]. 

**Figure 5:**
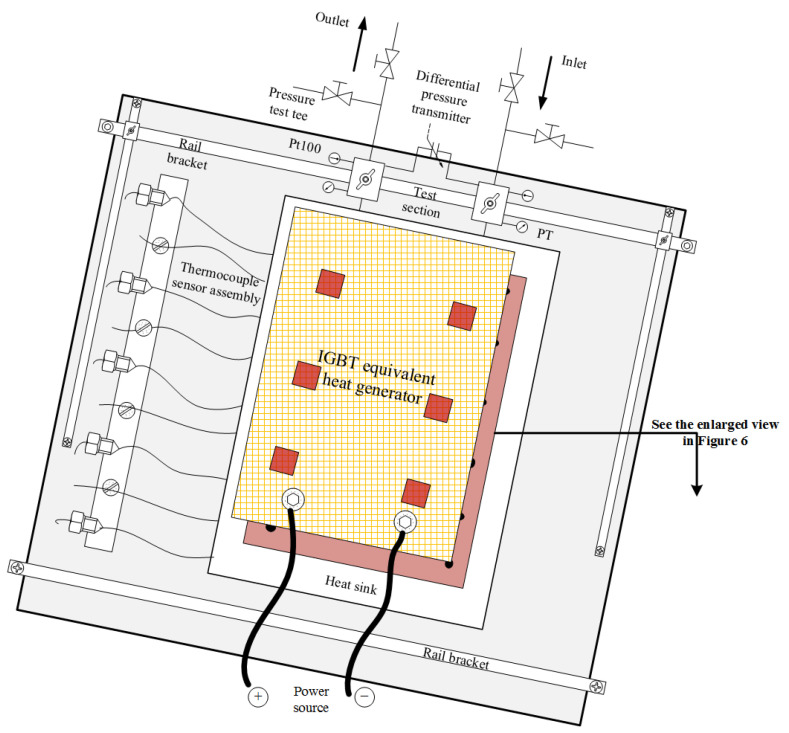
Testing platform designed for test section installation.

**Figure 6:**
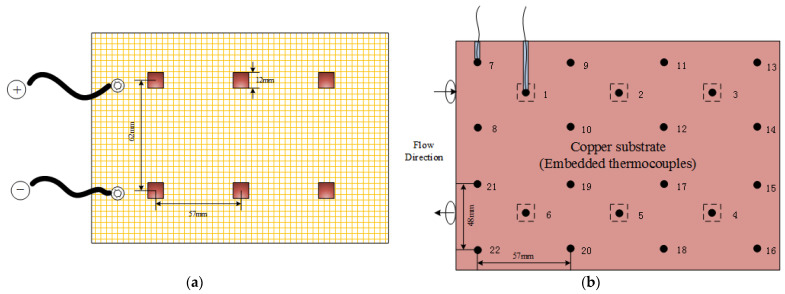
(**a**) Schematic diagram of the IGBT equivalent heat generator (**b**) Thermocouple arrangement.

Considering the high voltage level for an IGBT in actual operation and the requirements for adjusting heating power in experiments, an IGBT equivalent heat generator was applied, which is powered by a high precision DC power supply with maximum capacity of 60 V × 500 A. A special testing platform, which mainly consists of the rail brackets, adjustable working table panel, pressure test tees, bypass, sensor assembly and other accessories, was designed for easy installation of the test section and various measuring interfaces/devices, as shown in Figure 5. The equivalent heat generator has six hot spots, each with an area of 12 mm × 12 mm, which are used to simulate the performance of the IGBT heat producing process, as seen in Figure 6a.

In order to improve the measurement accuracy for temperature, a piece of counterbore copper substrate with high thermal conductivity is set between the equivalent heat generator surface and heat sink surface to bury and grip thermocouples tightly, which looks similar to a sandwich structure, as shown in Figure 5. The thickness of the copper substrate is about 2.5 mm, which is slightly larger than the diameter of thermocouple wire. Furthermore, both sides of the copper substrate contacting the heat transfer surfaces are coated with heat-conducting silicone grease. All these settings are to reduce thermal resistance caused by gaps between surfaces during the thermocouple installation. A total of 22 copper–constantan thermocouples (T-type), numbered 1–22, respectively, are uniformly distributed in the substrate. See Figure 6b for details. 

The transient heat transfer characteristics of high heat flux IGBT cooling is very important for the function performance and operation of electronic devices and systems. According to the IGBT technical features in Table 3, the maximum junction temperature is 125 °C. The IGBT shell temperature should be controlled under 100 °C, less than 25% of junction temperature, considering the body thermal resistance. Actually, the failure rate of electronic devices will double for every 10 °C increased from 65 °C, which is usually considered the safe temperature, and t is more likely to cause failure under off-normal situations [3,4,6]. Therefore, experiments were conducted especially for several abnormal conditions, such as instantaneous start-stop, transient heating power variation, and flow fluctuation. 

The procedure for experiments under instantaneous start-stop conditions is described as follows: Start the water circulating pump and the IGBT equivalent heat generator with constant flow rate and heating power, respectively. Wait until the system parameters are stable, cut off the power supply to stop the pump, but keep monitoring the temperature rise at all measuring points. Once any of the temperature points monitored have a high temperature (HT) over 80 °C, immediately put the circulating water pump into operation with the same flow rate before stopping. Adjust the heating power and flow rate to repeat the same procedure, and then obtain a series of results.

For transient heating power variation conditions, keep the system circulating normally with a constant flow rate and heating power, then suddenly increase the heating power until the high temperature phenomenon occurs. Immediately restore the heating power as before, continuously monitoring the temperature rise at all measuring points. Repeat the same procedure with different heating power and flow rates to get a series of results.

To observe the influence of flow fluctuation conditions on heat dissipation, keep the water circulating pump and the IGBT equivalent heat generator working normally, and stably collect system parameters, such as temperature, heating power, flow rate, etc., then adjust the flow fluctuation within a range 30% via the flow regulating valve. Keep the heating power unchanged while changing the flow rate and monitoring the temperature variation. According to different experimental requirements, the heating power and flow rate were adjusted, and the same process was repeated at different time intervals to obtain a range of results.

### 3.2. Data Reduction

The heating power *Q* of the IGBT equivalent heat generator is calculated by the high-precision DC power supply automatically recording the voltage *U* and current *I*.

(2) Q=(1−η)⋅ξ⋅P=(1−η)⋅ξ⋅U⋅I

where *η* is the heat loss coefficient and *ξ* is the power supply efficiency.

Transient heat transfer involving time should take account of time differential term, and thus the amount of heat transferred to cooling medium can be expressed as:

(3) $$Q=mC_{p}\frac{dT\left(t\right)}{dt}$$

where *m* is the mass flow of the cooling medium, *C_p_* is the specific heat capacity at constant pressure of the cooling medium, and *T* and *t* symbolize temperature and time, respectively.

As illustrated in the experimental scheme, copper substrate and thermal grease were used to reduce the thermal resistance due to gaps between the IGBT and heat sink. For the purpose of calculating the heat transfer coefficient, the average temperature value of the inner wall of the heat sink is derived by a one-dimensional steady heat conduction differential equation, which is considered as the multi-layer flat wall problem.

(4) $$\bar{T_{wi}}=\bar{T_{wo}}-Q\left(\frac{\delta_{1}}{A\lambda_{1}}+\frac{\delta_{2}}{A\lambda_{2}}+\frac{\delta_{3}}{A\lambda_{3}}\right)$$

where $\bar{T_{wo}}$ and $\bar{T_{wi}}$ are the average temperature of the heating surface, which is calculated according to the values of temperature measuring points, and the average temperature of the inner wall of the heat sink, respectively. The item $\delta_{i}/A\lambda_{i}$ in the bracket is the thermal resistance expression, and the subscripts *i* = 1, 2, 3 represent the copper base substrate, the silicone grease, and heat sink, respectively. *A* denotes the heat transfer area. In fact, the thermal resistance of a copper base substrate can be ignored because of its tiny thickness and high thermal conductivity.

The quasi equilibrium for heat transfer at time *t* can be written as:

(5) $$Q=Ah\left(\bar{T_{wi}}-T\left(t\right)\right)$$

where *h* is the convective heat transfer coefficient with time changing.

From Equations (3) and (5), equivalent transformation can be obtained as follows:

(6) $$mC_{p}\frac{dT\left(t\right)}{dt}=Ah\left(\bar{T_{wi}}-T\left(t\right)\right)$$

or

(7) $$\rho vC_{p}\frac{dT\left(t\right)}{dt}=Ah\left(\bar{T_{wi}}-T\left(t\right)\right)$$

where *ρ* and *v* are the density and specific volume of the working medium.

Equation (7) can be rearranged as:

(8) $$\frac{1}{\bar{T_{wi}}-T\left(t\right)}dT\left(t\right)=\frac{hA}{\rho vC_{p}}dt$$

The convective heat transfer coefficient *h* can be obtained in Equation (10) by integrating Equation (8) with integral limits in Equation (9).

(9) $$\int_{T_{1}}^{T_{2}}\frac{1}{\bar{T_{wi}}-T\left(t\right)}dT\left(t\right)=\int_{t_{1}}^{t_{2}}\frac{hA}{\rho vC_{p}}dt$$

(10) $$h=\frac{\rho vC_{p}}{A\left(t_{2}-t_{1}\right)}\ln\left(\frac{\bar{T_{wi}}-T_{2}}{\bar{T_{wi}}-T_{1}}\right)$$

The Reynolds number, Nusselt number and average Nusselt number are obtained according to the following formulas, respectively.

(11) $$\{\begin{array}{c} Re=\frac{u\cdot D_{h}}{\nu }\\ Nu=\frac{h\cdot D_{h}}{\lambda }\\ \bar{Nu}=\frac{\bar{h}\cdot D_{h}}{\lambda } \end{array}$$

where *u*, *v,* and *λ* are the velocity, the kinematic viscosity, and thermal conductivity of the working medium, respectively. The hydraulic diameter of a single flow channel is calculated by Equation (1), as described in Section 2.1. $\bar{h}$ is the average convective heat transfer coefficient calculated by:

(12) $$\bar{h}=\frac{\int_{t_{1}}^{t_{2}}hdt}{t_{2}-t_{1}}$$

### 3.3. Experimental Conditions and Uncertainties

To ensure the stability and accuracy of experiments, a pressure test and heat balance test were carefully conducted in advanced, using 99.99% high-pressure pure nitrogen and 60 mm thick glass fiber insulation material, respectively. The results show a 48 h pressure maintenance up to 4.2 MPa without leakage, and a heat loss of less than 5% at an average temperature of 200 °C. The repetition frequency of experiments depends on the specific parameters under the same conditions, but it must be ensured that the experimental data of each group should not deviate from the average value of all experiments by ±10%. Table 5 lists the ranges of the system parameters for current experiments. 

The inherent uncertainties of measuring instruments are determined according to the instructions and verified data sheet provided by the manufacturer. The maximum uncertainties in measuring channel size, temperature, pressure, flow rate, voltage, and current are ±0.096%, ±3.6%, ±2.0%, ±2.0%, ±1.5%, and ±2.5%, under the confidence levels of 96%, 97%, 98%, 99% and 98%, respectively. The average uncertainties for heat flux and heat transfer coefficient are ±4.4% and ±7.5% under the confidence levels of 96% and 95%, respectively. All these are calculated according to Moffat’s error analysis method in experimental measurements [46]. 

The uncertainty for a single direct measurement is expressed as:

(13) $$R\left(x_{i}\right)={\left(\delta x_{is}{}^{2}+\delta x_{ip}{}^{2}\right)}^{1/2}$$

where $\delta x_{is}$ is the system error of the measured value $x_{i}$; and $\delta x_{ip}$ is the random error of the measured value $x_{i}$.

For indirect measurement,

(14) $$X_{i}=f\left(x_{1},x_{2},\cdot \cdot \cdot ,x_{n}\right)$$

its uncertainty can be transferred by the following function:

(15) $$R\left(X_{i}\right)={\left(\overset{n}{\underset{i=1}{\sum }}{\left(\frac{\partial X_{i}}{\partial x_{i}}\delta x_{i}\right)}^{2}\right)}^{1/2}$$

## 4. Results and Discussion

### 4.1. Pre Experiments and Analysis

For the IGBT equivalent heat generator, which is described in Section 3.1 and Figure 6a, the six hot spots are considered as the heat generating switching devices inside IGBT. According to the actual operation performance and experimental requirements, at least three hot spots work at the same time with the same total power. Actually, the IGBT does not act that way exactly. The reason for setting hot spots in the current study is to take account of the influence of local high heat flux on heat dissipation. Thus, there are 42 kinds of heating combinations, calculated as:

(16) $$N=C_{6}^{6}+C_{6}^{5}+C_{6}^{4}+C_{6}^{3}=42$$

That is to say, three or more hot spots will share the total power, and will have different heat fluxes depending on the combination of hot spots. Therefore, in the current study the heat flux ranges from 104 W/cm^2^ to 347 W/cm^2^ when the heating power ranges from 900 W to 1500 W, which considers a range of ±25% variation in targeted IGBT heat generating power, i.e., 1200 W, as listed in Table 4. The switching behavior of 42 kinds of heating combinations is completely random, which is controlled by a programming compiler in IMPVIEW software.

**Figure 7:**
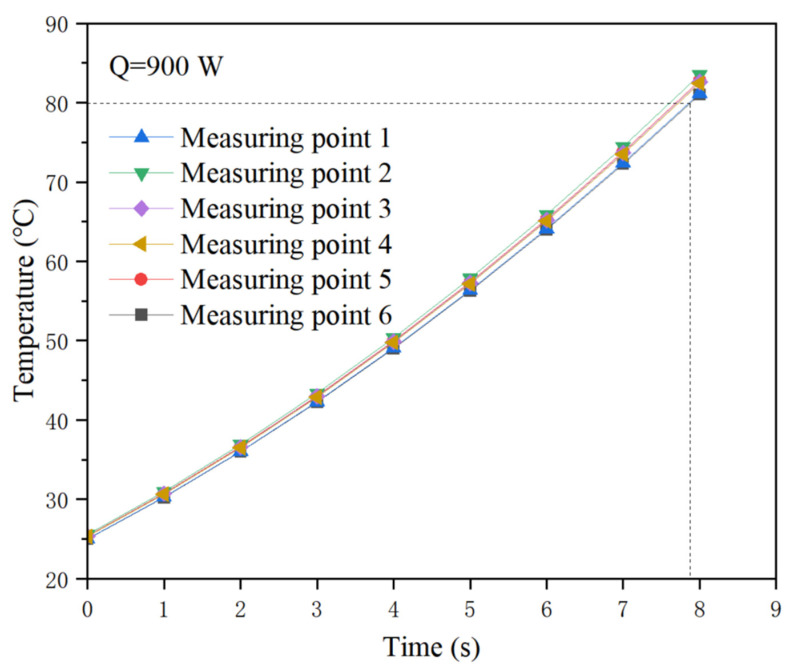
Temperature rise of measuring points without water cooling.

**Figure 8:**
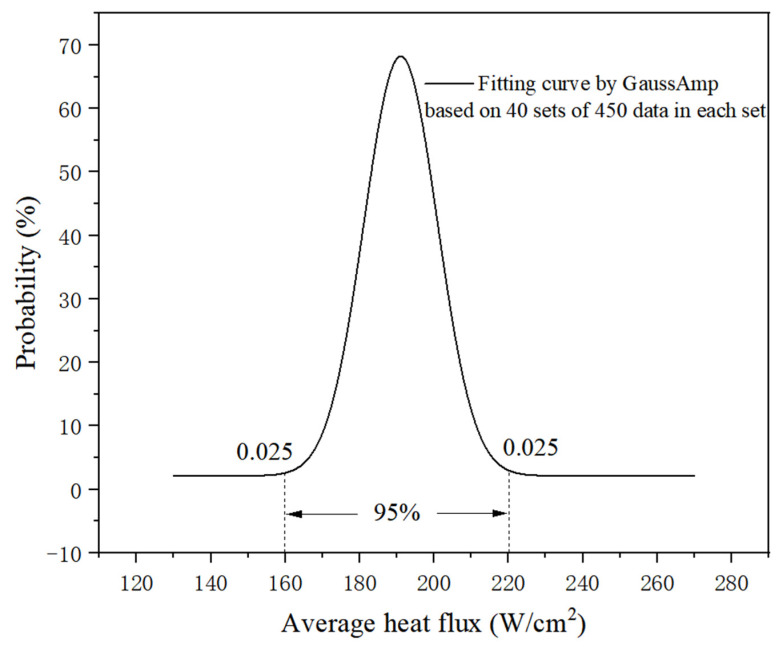
Probability statistics for random heating combinations.

Pre experiments were conducted to determine the working time for each heating combinations. The worst situation taken as an experimental condition is that the heating surface is cooled naturally without water cooling, under the conditions of six hot spots working simultaneously with a total heating power of 900 W. In this case, it will take more than 7 s to exceed 80 °C, as the results show in Figure 7. Therefore, it is reasonable to let each heating combination work for 8 s during each switching. Probability statistics for average heating power density are conducted to test the random heating combinations program and actual performance based on 40 sets of 450 data in each set, in which each switching lasts 8 s for an hour running under a total power of 1200 W. The result shows that the confidence level is 95%, as shown in Figure 8, covering the average heat flux from 160 W/cm^2^ to 221 W/cm^2^. Therefore, it is effective to use the average heat flux for heating power density evaluation.

### 4.2. Analysis of Instantaneous Start-Stop Conditions

According to the experimental procedure described in Section 3.1, the occurrence of high temperature is continuously monitored and the circulating pump is put into operation in a timely manner. The experiment is repeated with different parameters as needed. It is found that HT always occurs at the measuring points of 1–6 as expected, but it appears randomly. Under current experimental conditions, the temperature of the heating surface is not uniform, and the temperature of the hot spots are usually at a high temperature state, where the high heat flux concentrates on a small area. Table 6 lists the probability of HT occurrence at different positions based on 86 sets of experimental data.

From the table, HT appears at the measuring point positions 4–6 with higher probability than positions 1–3. The analysis shows that the residual liquid along the flow channel will evaporate or boil, taking away some of the heat. However, those positions where HT occurs are not cooled enough by residual liquid. The measuring points 2 and 5 have more frequency than others do in the same group of 1–3 or 4–6, respectively. The reason for this is that it is difficult for evaporation bubbles to escape in time due to the congestion in middle of channels without flow. Once there is fluid flow, this situation will be greatly improved.

**Figure 9:**
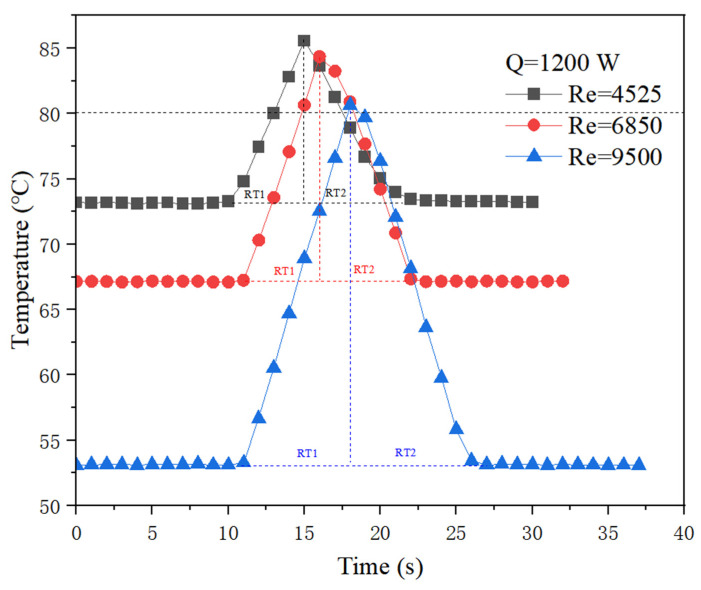
Temperature response characteristics to instantaneous start-stop conditions.

Experiments were carried out under instantaneous start-stop conditions with the typical heating power of 1200 W at various flow rates. Results are shown in Figure 9. It can be seen that the rates of three temperature rises are almost the same when water supply stops, but the cooling rate is obviously different under different flow rates when water supply is restored. The higher the flow rate the faster the cooling. To describe this difference, definition of RT1 and RT2 are applied to represent the respond time of instantaneous start-stop conditions. It can be seen that the RT1 segment is slightly shorter than the RT2 segment for all cases shown in Figure 9, which means the cooling rate is slower than the heating rate, although the flow rate restores the same as before stopping.

### 4.3. Analysis of Transient Heating Power Variation

To study the influence of transient heating power variation in the cooling effect, a range of results were obtained by following the experimental procedure described in Section 3.1.

**Figure 10:**
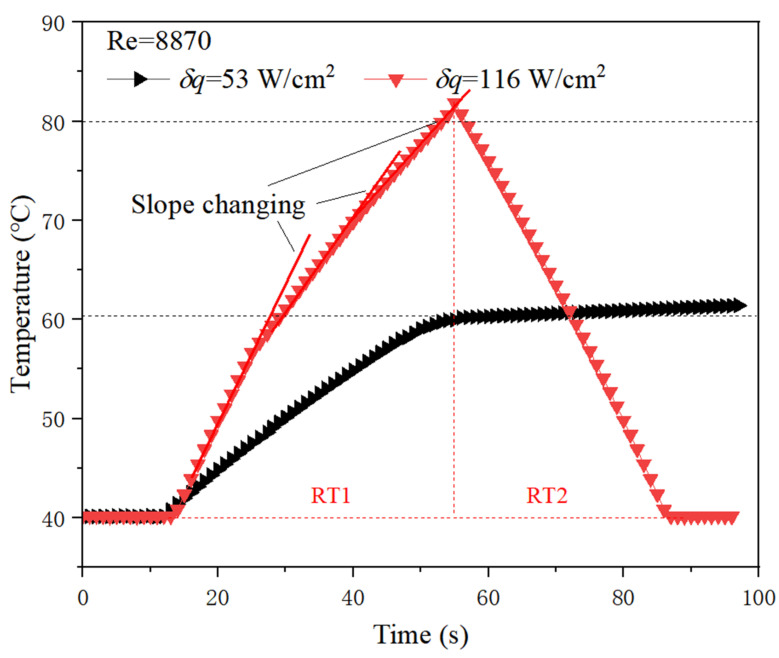
Temperature response characteristics to different heating power.

In Figure 10, *δq* represents the change in heat flux caused by transient heating power variation. From the figure, a small change in heating power (*δq* = 53 W/cm^2^) will not lead to a large temperature rise under relative large flow rate conditions, which can be seen from the black curve. When the heating power changes greatly (*δq* = 116 W/cm^2^), it may cause destructive temperature rise to an electronic device, which can be seen from the red curve. This also essentially indicates the limitation on the amount of heating power variation that can cause potential high temperature hazards at a given flow rate.

**Figure 11:**
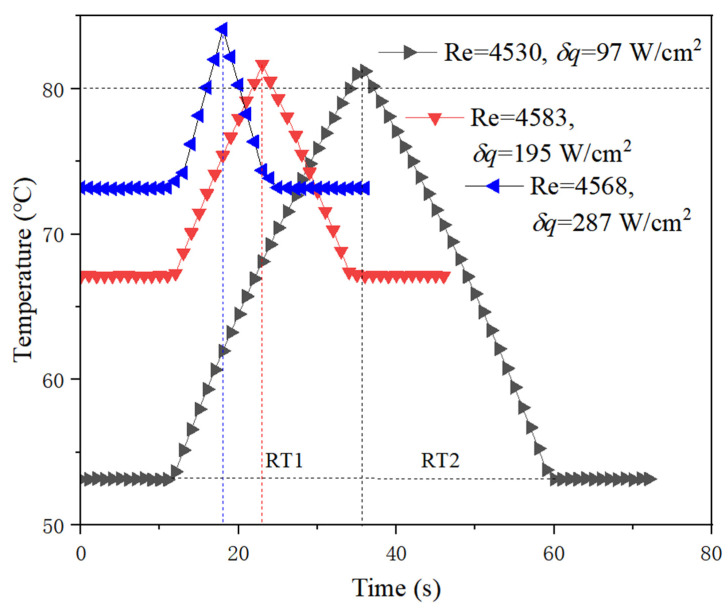
Temperature response characteristics at low flow rate.

**Figure 12:**
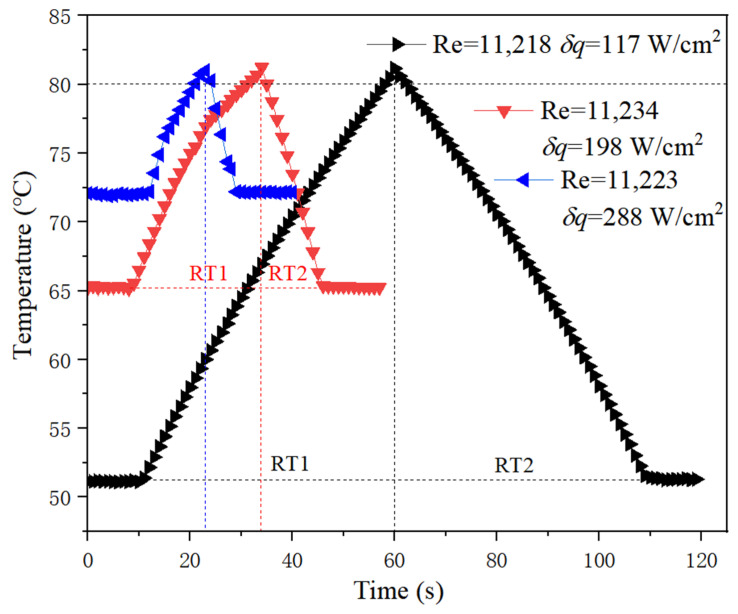
Temperature response characteristics at high flow rate.

For all the cases that HT occurs as shown in Figure 11 and Figure 12, the greater the increased heat flux, the faster the temperature exceeds HT value. At a low flow rate, i.e., low Re in Figure 11, RT1 and RT2 are almost equal, which indicates that the heating rate and cooling rate are basically the same. However, they are different at a high flow rate. Generally, the cooling rate is higher than the heating rate, as shown in Figure 12, that is to say, RT1 > RT2. Moreover, it can be seen that the slope for the heating curve is changing during the heating process and is decreasing with time, as indicated in Figure 10 in detail, which can be seen in Figure 12, as well at high flow Re over 11,000. Analysis reveals that the local subcooled boiling of liquid is the cause of the above phenomenon. Although the bulk working medium is in liquid state, local subcooling is unavoidable under high heat flux. If bubbles produced by subcooled boiling can be taken away in time, heat transfer will be enhanced, otherwise, it will be prevented. Therefore, high flow rate can reduce the rate of temperature rise by taking away the bubbles easily, while the effect of low flow rate is not obvious. However, this does not seem true for conditions represented by the black curve in Figure 12, although there is a high flow rate. It can be predicted that the subcooled boiling will not occur when the heat flux increment is not high under high flow rate, which does not slow the heating rate. Overall, under current experimental conditions, no heat transfer deterioration occurs. 

Through the above analysis, the comparison of RT1 and RT2 defined in this paper depends on different conditions. Respond time (RT) in Equation (17) is suggested to evaluate the transient condition of an unstable state for a given heating power increase, in which the influence of the difference between RT1 and RT2 on RT is considered. For safety assessment, it is reasonable to take the smaller value of RT1 and RT2 as the reference of RT, and the ratio of RT1 and RT2 is introduced as the correction factor to increase the margin, which reflects the relative effect of the cooling rate and heating rate. As indicated in Equation (17), this correction factor is equal to RT1/RT2 when Re < 4500, while it is equal to RT2/RT1 when Re > 8800. For 4500 < Re < 8800, it approximately equals 1. The greater the RT, the more time left for protecting the system, and the safer the system relatively is:

(17) $$\mathrm{RT}=\{\begin{array}{c} \frac{\mathrm{RT}1}{\mathrm{RT}2}\cdot RT1\;Re<4500\\ RT1\;4500<Re<8800\\ \frac{\mathrm{RT}2}{\mathrm{RT}1}\cdot RT2\;Re>8800 \end{array}$$

### 4.4. Analysis of Flow Fluctuation Conditions

**Figure 13:**
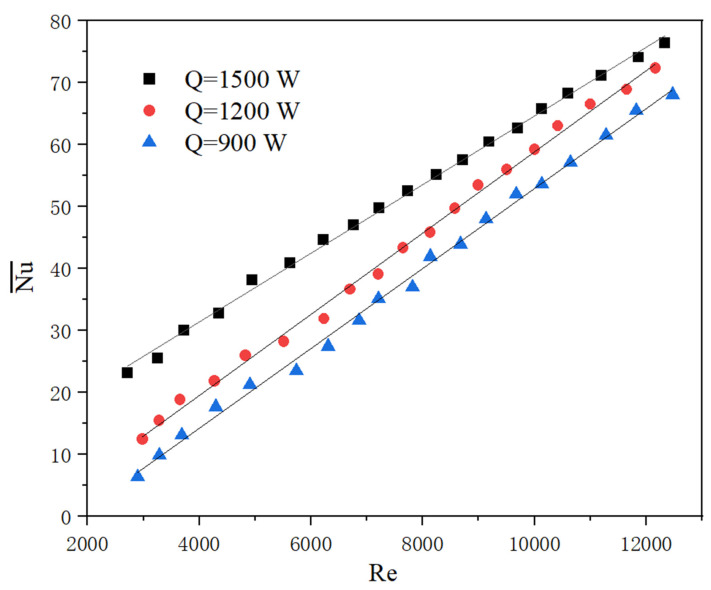
Nu variation with Re at different heating powers.

**Figure 14:**
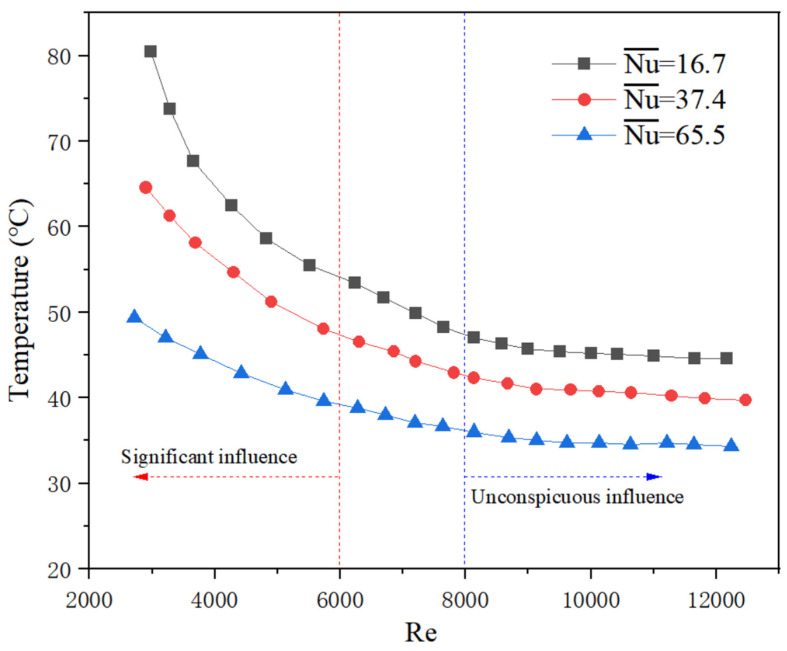
Temperature rise to flow fluctuation at different heat transfer intensity.

Generally, in practice when the flow fluctuates greatly, which is greater than 30%, the system will be shut down preferentially [2]. Since the situation of cutting off flow was discussed in Section 4.2, for the flow fluctuation, it is reasonable to take account of a fluctuation range of no more than 30%. Experimental results show that the average Nu varies with different Re, as shown in Figure 13, which is caused by different flow fluctuation, heating conditions, and physical properties. From Figure 14, it can be seen that the temperature rise rate increases significantly at a low flow rate (Re < 6000, as indicated on the left side of the red dash line). When the heat transfer intensity (represented by the average Nu number in the figure) is weak, a slight change in flow rate may result in a higher temperature rise at low Re. However, all the experiment data analysis indicates that the system with a stable state can be maintained at a range of safe temperature, which means this situation is not as dangerous as expected. At a high flow rate, the temperature rise rate is not obvious (Re > 8000, as indicated on the right side of the blue dash line).

In general, the flow fluctuation conditions are not as dangerous as instantaneous start-stop conditions that lead to the emergence situations, unless extreme heat transfer deterioration occurs or very high heat flux is applied.

## 5. Conclusions

In this paper, a flat plate heat sink made of aluminum for investigating the transient heat transfer characteristics of IGBT cooling was designed and manufactured. The influence of various abnormal conditions on the cooling performance was studied experimentally. The main conclusions are drawn as follows:

(1) The instantaneous start-stop conditions are very dangerous for IGBT cooling. The temperature measuring point located in middle of the channels is 20% more likely to have a high temperature than the side temperature measuring points. The heating rate is slightly higher than the cooling rate in all cases studied under these conditions. 

(2) Analysis of transient heating power variation indicates that the heating rate and cooling rate are basically the same at a low flow rate. However, they are different at a high flow rate. The temperature rise can be reduced under conditions of high flow rate and high heat flux increments. However, low flow rate does not act as effectively as high flow rate to reduce the heating rate. 

(3) The concept of respond time (RT) is suggested to evaluate the transient condition of an unstable state for the given increased heating power. The determinant of RT depends on different conditions listed in piecewise function, in which the smaller value of RT1 and RT2 is taken as the reference of RT, and the ratio of RT1 and RT2 is introduced as the correction factor to increase the margin, which reflects the relative effect of the cooling rate and heating rate.

(4) Experimental results under flow fluctuation conditions show that the average Nu varies with different Re, and flow fluctuation may cause a larger temperature rise at low Nu and Re. The temperature rise rate increases significantly when Re < 6000, while it is not obvious when Re > 8000.

## Figures and Tables

**Table 1:** Geometric parameters of the flow passageway.

Diameter of Inlet/mm	Diameter of Outlet/mm	Length of Single Mini-Channel/mm	Cross-Section Size/mm	Number of Flow Channels
12	12	249	3 × 4	20

**Table 2:** Classifications of flow channels based on *D_h_*.

Classifications	Mehendal et al. [43]	Kandlikar et al. [44]
Macro-channel	*D_h_* > 6 mm	*D_h_* > 3 mm
Mini-channel	100 μm < *D_h_* < 6 mm	200 μm < *D_h_* < 3 mm
Micro-channel	*D_h_* < 100 μm	*D_h_* < 200 μm

**Table 3:** Technical parameters of the Solartron IMP 3595 series.

Technical Parameters		IMP 3595 1A	IMP 3595 1C
Basic features	Number of channels	20	20
	Switching	solid-state, 3-pole	reed-relay, 3-pole
	Maximum signal measured	±12 V	±12 V
	Overload protection, continuous	50 V	50 V
	Maximum voltage between any input and any guard	14 V	200 V
	Common mode, between IMPs	500 V	500 V
Measurement	Voltage dc	0 to ±12 V	0 to ±12 V
	Current dc (assuming 100½ shunt)	0 to 20 mA	0 to 20 mA
	Thermocouple types	B, E, J, K, N, T, R, S	B, E, J, K, N, T, R, S
	Thermocouple cold junction	External or automatic	External or automatic
	Thermocouple open circuit detection	programmable	programmable

**Table 4:** Technical features of the IGBT module.

Manufacturer	Model	Electrical Features	Average Power Dissipation	Maximum Junction Temperature
Infineon^®^	FZ1500R33HE3	V_CES_ = 3300 V I_C, nom_ = 1500 A/I_CRM_ = 3000 A	1200 W	125 °C

**Table 5:** Experimental system parameters ranges.

System Parameters	Experimental Conditions		
	Instantaneous Start-Stop	Transient Heating Power Variation	Flow Fluctuation
Inlet temperature	25–35 °C	25–35 °C	30–35 °C
Flow rate	4.1–9.0 L/min	3.5–9.5 L/min	3.2–9.5 L/min
Heating power	960–1500 W	920–1500 W	900–1500 W
Heat flux of hot spot	120–298 W/cm^2^	112–316 W/cm^2^	104–347 W/cm^2^

**Table 6:** Distribution of high temperature at different positions.

Measuring Points	1	2	3	4	5	6
Frequency	10	13	12	15	19	17
Probability	11.6%	15.1%	13.9%	17.4%	22.1%	19.8%
